# Edmonton Frail Scale predicts mortality in older patients with cancer undergoing radiotherapy—A prospective observational study

**DOI:** 10.1371/journal.pone.0283507

**Published:** 2023-03-24

**Authors:** Inga Marie Røyset, Guro Falk Eriksen, Jūratė Šaltytė Benth, Ingvild Saltvedt, Bjørn Henning Grønberg, Siri Rostoft, Øyvind Kirkevold, Darryl Rolfson, Marit Slaaen

**Affiliations:** 1 Department of Neuromedicine and Movement Science, Faculty of Medicine and Health Science, NTNU—Norwegian University of Science and Technology, Trondheim, Norway; 2 Department of Geriatric Medicine, Clinic of Medicine, St. Olavs Hospital, Trondheim University Hospital, Trondheim, Norway; 3 The Research Centre for Age-Related Functional Decline and Disease, Innlandet Hospital Trust, Ottestad, Norway; 4 Institute of Clinical Medicine, Faculty of Medicine, University of Oslo, Oslo, Norway; 5 Department of Internal Medicine, Hamar Hospital, Innlandet Hospital Trust, Hamar, Norway; 6 Institute of Clinical Medicine, Campus Ahus, University of Oslo, Blindern, Norway; 7 Health Services Research Unit, Akershus University Hospital, Lørenskog, Norway; 8 Department of Clinical and Molecular Medicine, Faculty of Medicine and Health Science, NTNU—Norwegian University of Science and Technology, Trondheim, Norway; 9 Department of Oncology, St. Olavs Hospital, Trondheim University Hospital, Trondheim, Norway; 10 Department of Geriatric Medicine, Oslo University Hospital, Oslo, Norway; 11 Norwegian Advisory Unit on Ageing and Health, Vestfold Hospital Trust, Tønsberg, Norway; 12 Department of Health Sciences in Gjøvik, NTNU, Gjøvik, Norway; 13 Division of Geriatric Medicine, University of Alberta, Edmonton, Alberta, Canada; Cardiff University, UNITED KINGDOM

## Abstract

**Background:**

Several screening tools are developed to identify frailty in the increasing number of older patients with cancer. Edmonton Frail Scale (EFS) performs well in geriatric settings but is less studied in oncology. We aimed to investigate if EFS score (continuous and categorical) predicts survival in patients referred for radiotherapy, and to assess the concurrent validity of EFS compared with a modified geriatric assessment (mGA).

**Methods:**

Prospective observational, single-center study including patients ≥65 years, referred for curative or palliative radiotherapy for confirmed cancer. Patients underwent mGA (assessment of cognition, mobility, falls, comorbidity, polypharmacy, depression, nutrition, and activities of daily living) and screening with EFS prior to radiotherapy. The predictive value of EFS score of two-year overall survival (OS) was assessed by Kaplan-Meier plots and compared by log-rank test. Cox proportional hazards regression model was estimated to adjust the associations for major cancer-related factors. Concurrent validity of EFS in relation to mGA was estimated by Spearman`s correlation coefficient and ordinal regression. Sensitivity and specificity for different cut-offs was assessed.

**Results:**

Patients’ (n = 301) mean age was 73.6 (SD 6.3) years, 159 (52.8%) were men, 54% received curative-intent treatment, breast cancer (32%) was the most prevalent diagnosis. According to EFS≥6, 101 (33.7%) were classified as frail. EFS score was predictive of OS [hazard ratio (HR) 1.20 (95% confidence interval (CI) 1.10–1.30)], as was increasing severity assessed by categorical EFS (p<0.001). There was a strong correlation between EFS score and number of geriatric impairments (Spearman`s correlation coefficient 0.77). EFS cut-off ≥6 had a sensitivity of 0.97 and specificity of 0.57 for identifying patients with minimum two geriatric impairments.

**Conclusion:**

EFS predicts mortality in older patients with cancer receiving radiotherapy, and it is a quick (<5 minutes) and sensitive screening tool to identify patients who may benefit from a geriatric assessment.

## Introduction

Due to demographic changes, global cancer incidence is expected to increase by 47% in the next couple of decades [1], and more than two-thirds of cases are estimated to occur in older adults (age >65) [2]. This growing patient group is characterized by large heterogeneity in comorbidity burden and various degrees of functional decline. Related to this, the concept of frailty has gained increasing attention the last decades. Frailty is a geriatric syndrome characterized by increased vulnerability to stressors due to age-related declines in multiple organ systems [3, 4], resulting in increased risk of adverse outcomes across various clinical settings [4, 5]. Although numerous frailty criteria exist and there is no universally accepted definition [6], frailty is often classified on the basis of a geriatric assessment (GA) [7, 8]. GA is a comprehensive evaluation of health status by validated tools, assessing domains like functional status, physical performance, comorbidity (somatic, cognitive, mental), nutritional status and social support [9]. In patients with cancer, GA has a range of well-documented benefits, including identification of remediable impairments and prediction of survival and adverse events [9, 10]. Furthermore, GA may alter treatment decisions, facilitate shared decision making, improve patients’ satisfaction, and serve as a basis for targeted interventions and thereby improve outcomes [11–13].

Based on existing evidence, performing GA in older patients with cancer is thus widely recommended [9, 10, 14]. Despite this, GA has not been routinely implemented into clinical practice, and lack of resources in terms of knowledge, time, and trained staff have been recognized as important barriers [15, 16]. The use of a geriatric screening tool to select patients in need of a more thorough GA can be a pragmatic approach to this challenge [16–18]. Among several tools developed, the Geriatric-8 (G8) questionnaire [19] is currently the most frequently used and best validated in geriatric oncology [18, 20]. The Edmonton Frail Scale (EFS) is another brief screening tool, originally developed and validated in the geriatric setting [21]. This multidomain tool is quick (<5 minutes) with a good inter-rater reliability [21, 22], and in general surgical populations, frailty according to EFS is predictive of post-operative complications and longer hospitalization [22, 23]. To date, there is still limited data regarding the role of EFS in geriatric oncology. To address this issue, we investigate different properties of this tool in a cohort of older patients referred for radiotherapy (RT) due to cancer. In a previous publication based on the same patient cohort, we demonstrated that both specific GA domains and the number of geriatric impairments predicted overall survival (OS) [24]. In this paper, we specifically aim to investigate whether the EFS score (continuous and categorical) at baseline is predictive of two-year OS in this population; secondly, our objective is to assess the concurrent validity of EFS compared with a modified GA.

## Materials and methods

### Study design and participants

In this single-center prospective observational study, patients were consecutively recruited from the Radiotherapy Unit (RTU) at a Norwegian local hospital. Details on study setting, design and conduct have formerly been presented [24]. Inclusion criteria were age ≥65 years, referred for curative or palliative RT due to confirmed cancer (histology or cytology), ability to fill in questionnaires, and having a residential address in the catchment area (Innlandet County). Patients who the treating oncologist considered too sick for participation in the study were excluded. Apart from this, there were no specific exclusion criteria. All baseline registrations were performed by trained staff (the study nurse or a resident in oncology) prior to RT and the treating oncologist was blinded to the results of the study assessments. The patients were observed for survival for up to two years after RT completion, and information about time of death was retrieved from patients`hospital records.

### Baseline assessments

Cancer diagnosis according to The International Classification of Diseases, Tenth Revision (ICD-10), stage of disease (I-IV), Eastern Cooperative Oncology Group (ECOG) Performance Status (PS) and plan for RT (dose, field, curative vs. palliative intent) was registered from hospital records and information from the treating radiation oncologist. Cancer type was grouped into four categories: breast, prostate, lung, and other cancers, and ECOG PS was dichotomized to 0–1 vs. 2–4. All patients underwent frailty screening with EFS and a modified GA.

#### Edmonton Frail Scale

EFS covers nine domains including cognition, general health status, functional independence, social support, medication use, nutrition, mood, continence, and mobility. The score ranges from 0 to 17; higher score indicates higher levels of frailty. In the analyses, EFS was used both as a continuous and categorical variable. For the latter, patients were classified according to conventional EFS categories (edmontonfrailscale.org): fit (score of 0–3), vulnerable (4–5), mild frailty (6–7), moderate frailty (8–9), severe frailty (≥10).

#### Modified geriatric assessment

As the GA was performed by either a project nurse or a resident in oncology, not an interdisciplinary team, we have chosen to use the term *modified* GA (mGA) [25]. The mGA included nine domains, all assessed by widely used and well validated measures (Table 1). Comorbidity was measured with the Charlson Comorbidity Index (CCI) [26, 27] based on medical records and information from the patient. Cognitive function was evaluated with the Montreal Cognitive Assessment (MoCA) [28]. Regular medications were registered according to Anatomical Therapeutic Chemical (ATC) Classification System based on medical records or reports from patient or home nursing services. Mobility was assessed with Timed Up and Go (TUG) [29] and self-reported number of falls during the last six months. Activities of Daily Living (ADL) were registered with Barthel index [30], and Instrumental ADL (IADL) with Nottingham Extended Activities of Daily Living (NEADL) [31], both based on self-report. Nutritional status was measured according to the Mini Nutritional Assessment Short Form (MNA-SF) [32, 33]. Finally, depressive symptoms were assessed through patient interviews using the 15-item Geriatric Depression Scale (GDS-15) [34].

**Table 1:** Geriatric assessment: Domains, measures, and cut-off values for impairments.

Domain	Measure	Range	Interpretation	Cut-off for impairment	Rationale for cut-off
**Comorbidity**	Charlson Comorbidity Index (CCI)	0–26	Higher score = more comorbidity	≥2	Derived from Charlson et al. [26] and Sundararajan et al. [27]
**Medications**	Number of medications (ATC) taken regularly			≥5	Frequently used definition of polypharmacy [36, 37]
**Cognitive function**	Montreal Cognitive Assessment Test (MoCA)	0–30	Higher score = better cognitive function	65–75 years: ≤23, >75 years: ≤21	Derived from normative values from a large population study [35]
**Mobility**	Timed Up and Go (TUG)	Number of seconds		≥14 seconds	Previously used in cancer trials to identify GA deficits [37–40]
**Falls**	Number of falls the last six months			≥2	Previously used in cancer trials to identify GA deficits [38, 39]
**Activities of daily living (ADL)**	Barthel Index	0–20	Higher score = better function	≤18	Previously used in frailty classification of cancer patients [41]
**Instrumental activities of daily living (IADL)**	Nottingham Extended Activities of Daily Living (NEADL)	0–66	Higher score = better function	≤43	Previously used in frailty classification of cancer patients [41]
**Nutritional status**	Mini Nutritional Assessment Short Form (MNA-SF)	0–14 (Three categories: 0–7 = malnourished, 8–11 = at risk of malnutrition, 12–14 = normal nutritional status)	Higher score = better nutritional status	≤11	Cut-off for “at risk for malnutrition”. Validated in geriatric populations [33]
**Depressive symptoms**	Geriatric Depression Scale (GDS-15)	0–15	Higher score = more depressive symptoms	≥5	Most frequent cut-off value across different clinical settings [42]

For the analyses, and in accordance with a previous publication on this cohort [24], eight of the nine GA measures were dichotomized to define geriatric impairments. Established cut-off values were used when available (Table 1). As for cognitive function, where normative values are highly age-dependent [35], we chose two different cut-offs for the MoCA score according to age group, 65–75 years vs. >75 years.

### Statistical analysis

Descriptive data were reported as means with standard deviations (SDs) and median with minimum and maximum values for continuous variables, and frequencies and percentages for categorical variables. OS was defined as time from inclusion to death or last observation date (April 21, 2020). In the main, pre-planned analyses addressing the overall cohort, OS within the five conventional EFS categories was presented as Kaplan-Meier curves and compared by log-rank test. The predictive value of EFS (continuous score) on OS was estimated by Cox proportional hazards (PH) regression model, adjusting for established prognostic factors: age, gender, cancer type, treatment intent (palliative vs. curative), and ECOG status (model 1). Results were presented as hazard ratios (HRs) with corresponding 95% confidence intervals (CIs) and p-values. To further explore the impact of EFS scores on survival, OS was assessed by a similar Cox PH regression model omitting EFS (model 2), and another one, substituting EFS with number mGA impairments (model 3). C-indices, assessing discriminative ability of the models, were calculated and compared between the three models. Finally, post hoc, and for explorative purposes, we estimated similar models stratified by treatment intent.

We also investigated if alternative EFS categories based on different cut-offs could improve categorization of patients with respect to survival. For this, based on martingale residual plot with lowess smoother, we chose 17 different sets with cut-offs including two to nine different EFS cut-off values, resulting into three to ten frailty categories. For each set, we estimated Cox PH model and assessed its fit by four statistical criteria: log-likelihood (higher means better), log-rank (higher means better), Bayes Information Criterion (BIC) and Akaike’s Information Criterion (AIC) (lower means better). The C-index was compared between the Cox PH model with EFS categorized to five conventional and the Cox PH model with EFS categorized by identified alternative cut-offs.

The prevalence of mGA impairments (according to the defined cut-off values of each measure) were estimated within each conventional EFS category and presented as frequencies and percentages. The association between EFS categories and the number of -mGA impairments was quantified by Spearman`s correlation coefficient and ordinal logistic regression analysis with EFS as independent and mGA impairments as dependent variable, with results presented as odds ratios (ORs) and 95% CIs. The association between the two variables was visualized by box plot.

Finally, we performed additional analyses to evaluate EFS’s ability to detect different number of mGA impairments. EFS was dichotomized at all potential cut-offs (non-frail vs. frail), and sensitivity and specificity were assessed with respect to different numbers of mGA impairments (min-max 0–9). Area under the curve (AUC) was also estimated for all EFS cut-off values and presented with corresponding 95% CI.

Model assumptions were assessed by standard methods. SPSS Statistics version 26 (IBM Corp. Released 2019. IBM SPSS Statistics for Windows, Version 26.0. Armonk, NY: IBM Corp) and Stata version 16 (StataCorp. 2019. Stata Statistical Software: Release 16. College Station, TX: StataCorp LLC) were used for statistical analyses. Statistical significance level was defined as 0.05.

### Ethics and approval

Written informed consent was obtained from all patients. The Regional Committee for Medical and Health Research Ethics South-East Norway approved the study protocol (2016/2031/REK sør-øst), and the study was registered in ClinicalTrials.gov (NCT03071640).

## Results

### Recruitment and patient characteristics

Between February 2017 and July 2018, 538 patients were screened for inclusion. Of 509 eligible patients 301 (59.1%) were included in the study. The remaining were not included as they either declined participation (n = 148, 29.1%), were considered too sick (n = 28, 5.5%), or had other reasons such as an absent study nurse (n = 32, 6.3%).

An overview of baseline characteristics is given in Table 2. Mean age was 73.6 (SD 6.3) years, 159 (52.8%) were men, and 162 (53.8%) received treatment with curative intent. The most prevalent cancer diagnoses were breast (n = 95, 31.6%), prostate (n = 73, 24.3%) and lung (n = 65, 21.6%). Median EFS score was 4 (range 0–12). Approximately one-third (n = 101, 33.6%) of the patients were classified as frail according to EFS score ≥6. In this group there were 58 (57.4%) men. Lung cancer was the most prevalent cancer type (n = 34, 33.7%), and the majority were in a palliative setting (n = 76, 75.2%). The distribution of EFS categories within the overall cohort and in groups defined according to treatment intent is presented in Table 3.

**Table 2:** Baseline characteristics.

	All patients (n = 301)	Frail, EFS ≥6 (n = 101)	Non-frail, EFS <6 (n = 199)
**Age**			
Mean (SD)	73.6 (6.3)	75.6 (7.1)	72.6 (5.7)
Median (min-max)	72 (65–96)	74 (65–96)	71 (65–89)
**EFS**			
Mean (SD)	4.5 (3.0)	8.2 (1.7)	2.7 (1.5)
Median (min-max)	4 (0–12)	8 (6–12)	3 (0–5)
**Gender,** n (%)			
Female	142 (47.2)	43 (42.6)	99 (49.7)
**Cancer type**, n (%)			
Breast (C50)	95 (31.6)	22 (21.8)	73 (36.7)
Prostate (C61)	73 (24.3)	18 (17.8)	55 (27.6)
Lung (C34)	65 (21.6)	34 (33.7)	30 (15.1)
Other	68 (22.6)	27 (26.7)	41 (20.6)
**Stage**, n (%)			
I	62 (20.6)	9 (8.9)	53 (26.6)
II	42 (14.0)	11 (10.9)	31 (15.6)
III	78 (25.9)	16 (15.8)	61 (30.7)
IV	119 (39.5)	65 (64.4)	54 (27.1)
**Treatment intent**, n (%)			
Curative	162 (53.8)	25 (24.8)	137 (68.8)
Palliative	139 (46.2)	76 (75.2)	62 (31.2)
**ECOG PS**, n (%)			
0–1	256 (85.0)	57 (56.4)	199 (100)
2–4	45 (15.0)	44 (43.6)	0 (0.0)
**Impairments of mGA** *			
Comorbidity (CCI)	82 (27.2)	46 (45.5)	35 (17.6)
Polypharmacy	166 (55.1)	86 (85.1)	79 (39.7)
Cognitive impairment (MoCA), 65–75 years	66 (33.3)	28 (49.1)	38 (26.6)
Cognitive impairment (MoCA), > 75 years	39 (39,0)	27 (61.4)	12 (21.4)
Mobility (TUG)	64 (21.3)	52 (51.5)	11 (5.5)
Falls	34 (11.3)	17 (16.8)	17 (8.5)
ADL (Barthel index)	60 (19.9)	52 (51.5)	7 (3.5)
IADL (NEADL)	63 (21.1)	59 (59.6)	3 (1.5)
Nutritional status (MNA-SF)	167 (55.5)	89 (88.1)	77 (38.7)
Depressive symptoms (GDS-15)	63 (20.9)	45 (44.6)	17 (8.5)

*N = 300 for all groups except MoCA 65–75 years (N = 198), MoCA >75 years (N = 100), NEADL (N = 297).

Abbreviations: EFS, Edmonton Frail Scale; ECOG PS, Eastern Cooperative Oncology Group Performance Status; mGA, modified Geriatric Assessment; CCI, Charlson Comorbidity Index; MoCA, Montreal Cognitive Assessment; TUG, Timed Up and Go; ADL, Activities of Daily Living; IADL, Instrumental ADL; NEADL, Nottingham Extended Activities of Daily Living; MNA-SF, Mini nutritional Assessment Short Form; GDS-15, 15-item Geriatric Depression Scale.

**Table 3:** Distribution of EFS categories.

	Overall cohort	Treated with curative intent	Treated with palliative intent
EFS categories	N (%)	N (%)	N (%)
Fit (0–3)	134 (44.5)	101 (62.3)	33 (23.7)
Vulnerable (4–5)	65 (21.6)	36 (22.2)	29 (20.9)
Mild Frailty (6–7)	39 (13.0)	9 (5.6)	30 (21.6)
Moderate Frailty (8–9)	39 (13.0)	11 (6.8)	28 (20.1)
Severe Frailty (10+)	23 (7.7)	5 (3.1)	18 (12.9)
Missing	1 (0.3)	0 (0)	1 (0.7)
Total	300	162	139

### EFS in relation to overall survival

In the entire cohort, 123 (40.9%) died within the two-year follow-up. Of these 120 (89.4%) and 13 (10.6%) received treatment with palliative and curative intent, respectively. In the frail group (EFS ≥6), 68 (67.3%) patients died, opposed to 54 (27.1%) in the non-frail group (EFS <6). Median observation time for the overall cohort was 24.2 months (min 0.3, max 25.9).

**Fig 1:**
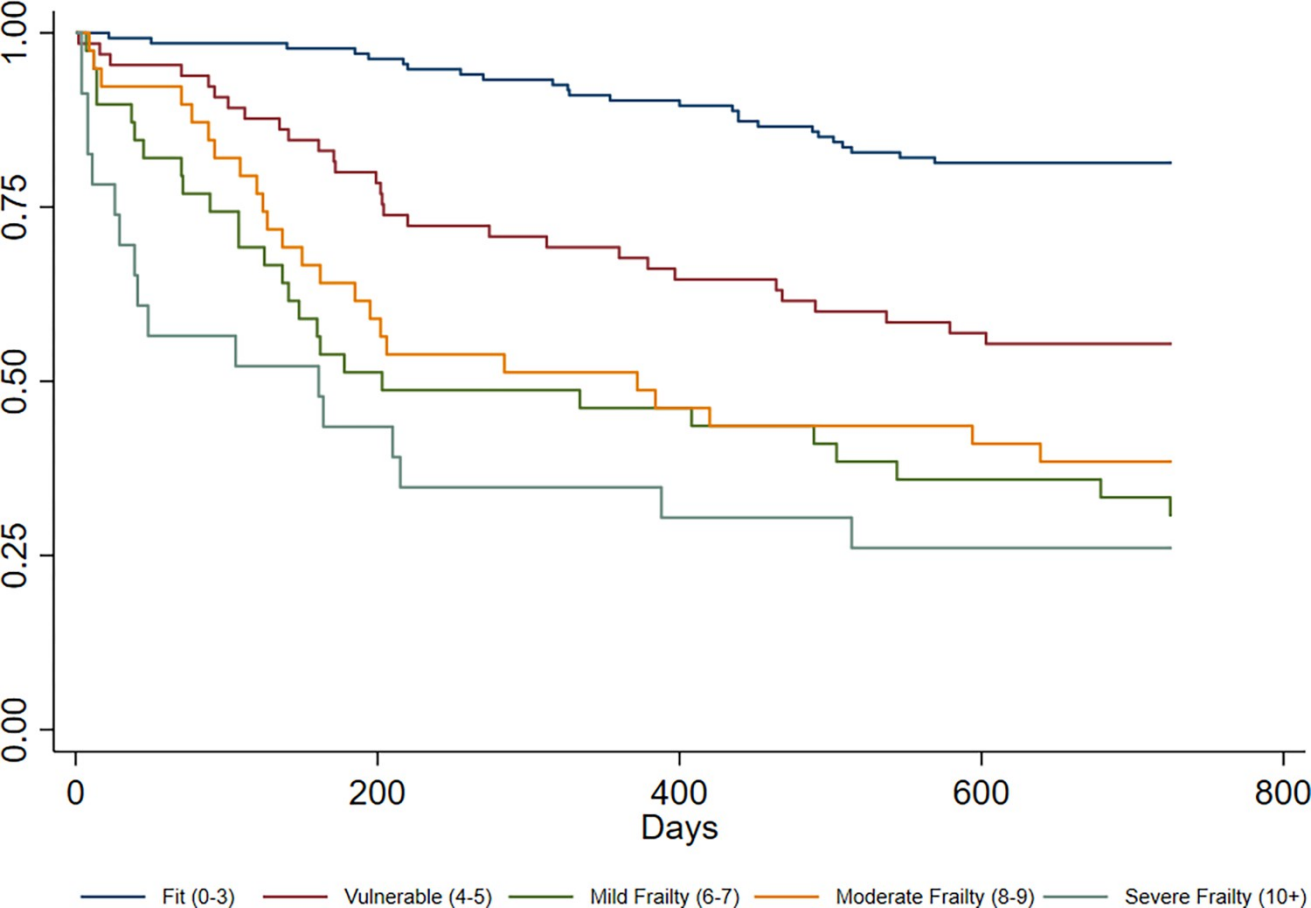
Kaplan-Meier survival curves for overall survival (days) according to conventional EFS categories.

OS stratified by the five conventional EFS categories is presented as Kaplan-Meier curves in Fig 1. Patients categorized as fit (EFS score 0–3) had the best OS, and OS declined with higher EFS score (i.e., higher levels of frailty). According to log-rank test, there were significant differences in OS between groups (p<0.001).

According to bivariate Cox regression analysis, increase in EFS score at baseline was significantly associated with worse two-year OS [HR 1.27 (95% CI 1.20; 1.35, p<0.001)] (Table 4). The association remained significant after adjustment for age, sex, treatment intention, cancer type, and ECOG-status [HR 1.20 (95% CI 1.10; 1.30, p<0.001)]. Thus, a one-point increase in baseline EFS score led to a 20% increased risk of dying within two years (Table 4, multiple model 1, main). Additionally, receiving palliative treatment and having lung cancer significantly increased this risk. Similar results for receiving palliative treatment were found in the explorative models, both the model where EFS scores were excluded (Table 4, model 2) and the model where EFS scores were substituted by number of mGA impairments (Table 4, model 3). According to model 3, increasing number of mGA impairments were independently associated with worse OS [HR 1.23 (95% CI 1.10; 1.38, p<0.001)], meaning that for each impairment added, the risk of dying increased by 23% (Table 4, model 3). The C-indices for model 1, 2 and 3 were 0.851, 0.839 and 0.848, respectively (Table 4), and did not differ significantly between model 1 and 2 (p = 0.101), model 1 and 3 (p = 0.508) or model 2 and 3 (p = 0.179).

**Table 4:** Cox proportional hazard models estimating the relationship between covariates and overall survival.

	Bivariate models		Multiple model 1 (main)		Multiple model 2		Multiple model 3	
Covariates	HR (95% CI)	p-value	HR (95% CI)	p-value	HR (95% CI)	p-value	HR (95% CI)	p-value
EFS score	1.27 (1.20; 1.35)	**<0.001**	1.20 (1.10; 1.30)	**<0.001**	**-**	**-**	**-**	**-**
N° of geriatric impairments					-	-	1.23 (1.10; 1.38)	**<0.001**
Age	1.04 (1.02; 1.07)	**0.002**	1.00 (0.97; 1.03)	0.995	1.01 (0.98;1.04)	0.484	1.01 (0.98; 1.04)	0.497
Gender (female)	0.57 (0.40; 0.82)	**0.003**	0.80 (0.52; 1.24)	0.319	0.91 (0.59; 1.39)	0.649	0.73 (0.47; 1.14)	0.170
Treatment intent (palliative)	18.93 (10.60; 33.80)	**<0.001**	9.43 (4.96; 17.95)	**<0.001**	11.57 (5.99; 22.35)	**<0.001**	10.97 (5.75; 20.91)	**<0.001**
Cancer type								
Breast	1		1		1		1	
Prostate	2.21 (1.04; 4.68)	**0.038**	0.93 (0.37; 2.32)	0.877	0.93 (0.37; 2.30)	0.871	0.75 (0.30; 1.87)	0.539
Lung	11.36 (5.88; 21.93)	**<0.001**	2.52 (1.17; 5.46)	**0.019**	2.19 (1.00; 4.79)	0.050	1.87 (0.86; 4.10)	0.116
Other	8.60 (4.43; 16.66)	**<0.001**	1.78 (0.82; 3.87)	0.144	1.63 (0.74; 3.58)	0.224	1.38 (0.63; 3.03)	0.417
ECOG dichotomized (2–4)	3.57 (2.38; 5.36)	**<0.001**	0.87 (0.51; 1.46)	0.589	1.83 (1.21; 2.77)	**0.004**	0.73 (0.39; 1.37)	0.333
C-index (95% CI)			0.851 (0.821; 0.881)		0.839 (0.807; 0.871)		0.848 (0.817; 0.878)	

Abbreviations: EFS, Edmonton Frail Scale; ECOG, Eastern Cooperative Oncology Group.

Multiple Cox regression models, stratified by treatment intent, confirmed the statistically significant, independent predictive value of EFS scores in the group receiving RT with palliative intent [HR 1.17 (95% CI 1.06; 1.28) (p = 0.001)] (S1 Table). In patients who received treatment with curative intent, EFS scores were not associated with OS [HR 1.10 (95% CI 0.89; 1.37) (p = 0.370) (S1 Table). The only significant, predictive factor was having lung cancer [HR 50.60 (5.43; 471.79) (p = 0.001)].

When exploring if alternative EFS cut-offs could improve categorization with respect to survival in the overall cohort, we found that a model with seven categories (EFS score 0–2, 3, 4–5, 6–7, 8–9, 10, ≥11) (unadjusted for other covariates) had a C-index of 0.73 (95% CI 0.69; 0.77), compared to 0.72 (95% CI 0.68; 0.76) for the conventional five category model. The difference between these models was not statistically significant (p = 0.109).

### Concurrent validity of EFS to mGA findings

**Fig 2:**
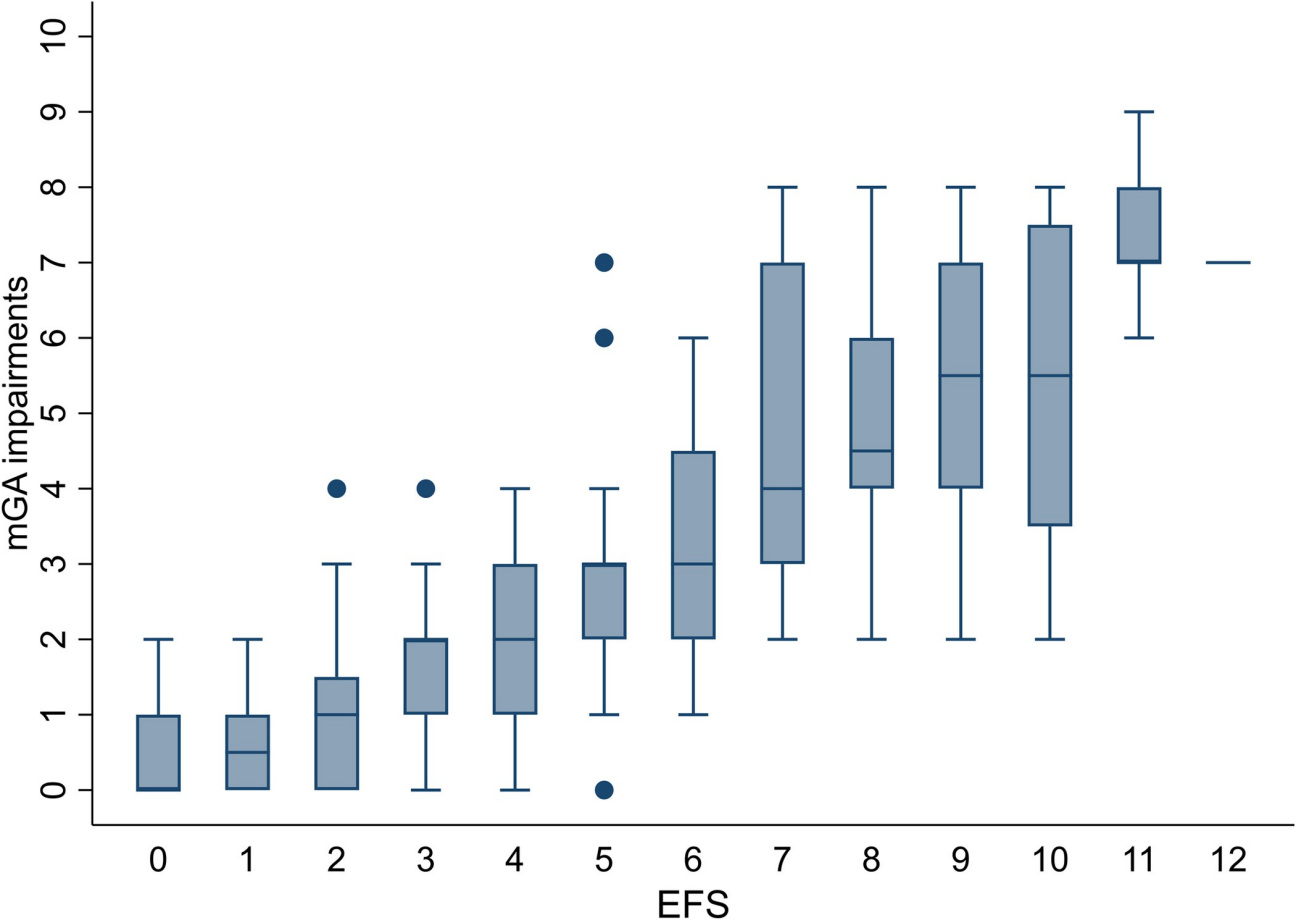
Box plot demonstrating the correlation between EFS scores and number of mGA impairments.

The number of mGA impairments varied from zero to nine; 49 patients (16.3%) had none and 81 (26.9%) had at least four impairments. The prevalence of mGA impairments within groups defined by conventional EFS categories is presented in Table 5. We found a strong association between increasing frailty according to EFS category and increasing number of mGA impairments (Spearman’s correlation coefficient 0.77), visualized by a box plot in Fig 2. Similarly, increasing frailty according to EFS category increased the odds for having a higher number of mGA impairments. Compared to patients with severe frailty, the odds were statistically significantly lower for patients categorized as fit, vulnerable, or mildly frail, but not for those categorized as moderately frail (Table 4, right column).

**Table 5:** Prevalence of mGA impairments according to EFS category, and ordinal logistic regression analysis.

EFS categories	Number of mGA impairments (%)					Ordinal regression analysis	
	0	1	2	3	≥4	OR (95% CI)	p-value
Fit (0–3)(n = 134)	46 (34.6)	52 (39.1)	22 (16.5)	11 (8.3)	2 (1.5)	0.002 (0.000–0.011)	<0.001
Vulnerable (4–5) (n = 65)	3 (4.7)	12 (18.8)	18 (28.1)	22 (34.4)	9 (14.1)	0.021 (0.005–0.098)	<0.001
Mild Frailty (6–7) (n = 39)	0	33 (7.9)	6 (15.8)	8 (21.1)	21 (55.3)	0.110 (0.023; 0.526)	0.006
Moderate Frailty (8–9) (n = 39)	0	0	3 (7.9)	6 (15.8)	29 (76.3)	0.327 (0.065; 1.652)	0.176
Severe Frailty (10+) (N = 23)	0	0	1 (4.5)	1 (4.5)	20 (90.2)	1	-
Total	49	67	50	48	81		

Abbreviations: EFS, Edmonton Frail Scale; mGA, modified Geriatric Assessment. Number of mGA impairments as dependent variable and EFS categories as independent variable.

Using the conventional cut-off for frailty (EFS ≥6), we found a sensitivity of 0.97 and a specificity of 0.57 for identifying patients with at least two GA impairments, and 0.87 and 0.78 for identifying at least three GA impairments, respectively. By reducing the EFS cut-off with one point to ≥5, we found a sensitivity of 0.95 and specificity 0.65 for identifying at least two impairments, and 0.80 and 0.83 for minimum three impairments, respectively. By increasing the EFS cut-off to ≥7, corresponding numbers for sensitivity was 1.00 and 0.92 for identifying minimum two and three impairments, and specificity was 0.54 and 0.74 for identifying minimum two and three impairments. Area under the curve (AUC) was 0.91 (95% CI 0.88; 0.94) for EFS cut-off ≥5, 0.92 (95% CI 0.89; 0.95) for cut-off ≥6, and 0.94 (95% CI 0.91; 0.96) for cut-off ≥7. An overview of sensitivity analyses for all EFS cut-off values in relation to different number of mGA impairments is presented in S2 Table.

## Discussion

In this observational study, we investigated different properties of EFS in a cohort of older patients with cancer referred for RT with either palliative or curative intent. We found EFS score to be an independent predictor of two-year OS. There were also significant differences in OS according to conventional EFS categories. Further, increasing frailty measured with EFS was strongly correlated to increasing number of mGA impairments. Using the cut-off EFS ≥6, we found a high sensitivity and a moderate specificity for identifying patients with at least two GA impairments.

To the best of our knowledge, only a couple of smaller studies have previously looked at the association between EFS scores and OS in older patients with cancer [43, 44]. Meyers et al. [43] found a negative relationship between EFS ≥7 and OS among 46 patients with colorectal cancer, whereas in a study by Richards et al. following 86 patients with the same diagnosis [44], no conclusion could be drawn due to few deaths during the study period. Our findings are however, supported by larger studies from older non-cancer populations, showing that EFS scores are predictive of OS [45–47]. Furthermore, two of these studies [45, 46], reported significant differences in OS according to frailty categories (EFS scores 0–3, 4–6 and ≥7). Our study expands this knowledge, indicating that in older patients with cancer, the conventional EFS categories differentiate between five distinctly different groups in terms of survival, similar to what can be achieved by classification according to number of GA impairments [24]. Furthermore, our findings indicate that EFS scores have a predictive strength comparable to the count of such impairments. For confirmation, and to evaluate EFS’s ability to provide prognostic information related to other outcomes in the RT setting, future studies are needed. Hitherto, the only study investigating the relationship between EFS and RT toxicity [48] was negative, implying that the increased mortality in patients with a higher degree of frailty may not be treatment-related.

When comparing predictive models, we found that neither EFS scores nor frequency of geriatric impairments seemed to add information to established cancer related prognostic factors. Even if confirmed, this finding cannot serve as an argument against performing GA, which in older patients with cancer has a range of other well-documented benefits [11, 12]. Regarding the use of EFS, our results indicate that the instrument may provide a finely tuned prognostic tool, which could be particularly useful for treatment decisions in routine clinical practice. More importantly, one should keep in mind that the main purpose of frailty screening is not to predict survival, but to identify patients who will profit from a full GA. In this respect, the present study indicates that EFS is a relevant instrument to apply in older patients with cancer referred for RT.

We found a strong correlation between higher EFS scores and increasing number of geriatric impairments, and demonstrated that with a sensitivity of 0.97, the EFS cut-off ≥6 will capture almost all patients with at least two geriatric impairments, although the lower specificity (0.57) means that 43% will be classified as false positive. We are not aware of comparable studies from a cancer setting, but in line with the present findings, Perna et al. [49] found significant associations between EFS and several geriatric domains in a cohort of hospitalized older adults. Furthermore, the diagnostic accuracy of G8, the most widely used screening tool for frailty in geriatric oncology, is reportedly in line with our results for EFS. A systematic review revealed a median sensitivity for G8 in identifying frailty of 85% (range 38%-97%) and a specificity of 64% (range 28%-100%) across 19 studies [8]. In all studies, frailty was identified by GA, and consistent with the present study, the majority used a cut-off of two impairments or more [8]. In comparison to G8 [19], EFS presents the advantage of including tests for cognitive function (clock-drawing) and physical performance (TUG), and mapping of social support [21]. Thus, if comparable properties are confirmed in future studies, we believe that EFS should be included in the toolbox for frailty screening in patients with cancer.

This study has some limitations and our results must be interpreted with these in mind. A main point to consider is the heterogeneity of our study sample in terms of demographics, general health status, cancer type, and treatment intent. This can be seen as a study strength, implying that our results are relevant for routine oncology clinics, and consequently that EFS performs well and holds prognostic information in a diverse group of patients. On the other hand, the heterogeneity introduces the likelihood of unaddressed confounders. In our pre-planned survival analyses, we included major, established prognostic factors, but due to a relatively limited sample size, the number had to be restricted to retain statistical power. We chose, for instance, to include treatment intent at the expense of stage of disease. The two variables were strongly correlated, and we believe that treatment intent as decided by the treating oncologist holds the most essential prognostic information in a heterogeneous cohort like the present one. Overall, however, we cannot rule out that known and unknown factors other than those taken into account, may have influenced our results. Furthermore, patients receiving treatment with curative and palliative intent are obviously clinically distinct. We therefore performed post hoc analyses on a stratified study sample. The independent predictive value of EFS scores was reproduced in the sub-cohort receiving treatment with palliative intent, but not among those receiving curative RT. In this latter group, only 13 patients died during the follow-up, and the distribution of EFS scores was highly skewed towards the fit category. For these reasons, as well as smaller sample size resulting from dividing the overall cohort and the exploratory nature of the analyses, the results must be interpreted with caution. Thus, future studies are needed to establish the prognostic value of EFS in more homogeneous samples in terms treatment intent, type of cancer and stage of disease.

Other limitations of our study are firstly the truncated range of EFS scores and a potential selection bias. The upper EFS limit is 17, whereas in our cohort the highest score was 12. This may have influenced our validation analyses. Moreover, the overall skewness of scores towards the fitter range, together with relatively large proportion of non-included eligible patients, suggest that the fitter patients might have been selected for enrolment. It may, however, also reflect that many frail patients are not referred for RT. Secondly, due to limited resources, both EFS and mGA were performed by the same assessor. This may potentially lead to overestimation in the concurrent validity analyses. We do however, find this unlikely. By the time of assessment, the assessors had no knowledge of the definition of geriatric impairment, the cut-offs for EFS categories, or how the validation would be performed. To validate EFS as a screening tool, we used a frequently applied approach, that is, evaluation against GA [18]. A common challenge for such studies is the lack of consensus on how to identify frailty based on GA. Consequently, procedures vary between studies, making comparison of results difficult [7, 8, 18]. We chose to evaluate EFS against the number of geriatric impairments, and this we consider as a study strength. Additionally, our main focus was the accuracy of the conventional EFS cut off for frailty (≥ 6) in identifying patients with two or more geriatric impairments, the most commonly used cut off for frailty based on GA [7, 50]. The content of our mGA is also a study strength. The comprehensive assessment of nine geriatric domains with well-validated tools is in accordance with ASCO Guideline [9], and cut-offs for impairments were based on previously used cut-offs when available. Finally, in the Cox regression models estimating EFS prognostic ability, we followed strong recommendations for studies on prognostic factors, and included EFS as a continuous variable, not categorized [51].

In conclusion, we found EFS to be an independent predictor of OS in a heterogeneous population of older adults with cancer referred for RT, and demonstrated that EFS has a high sensitivity in identifying patients with geriatric impairments who are in need of a more elaborate GA. Adding that EFS is quick (< 5 minutes) and covers multiple geriatric domains, allowing for a subsequent focused assessment of the particular components of interest, it appears to be a topical tool for frailty screening in older patients with cancer receiving RT. Future studies addressing EFS’ performance in more homogeneous cancer populations are warranted.

## Supporting information

S1 Table Cox regression models estimating the relationship between EFS score and OS in patients stratified by curative and palliative intent. (DOCX)

S2 Table Sensitivity, specificity, and area under the curve (AUC) for different EFS cut-offs in relation to different numbers of mGA impairments. (DOCX)
