# Supplementary material for: Edmonton Frail Scale predicts mortality in older patients with cancer undergoing radiotherapy—A prospective observational study
Source: PLoS One. 2023 Mar 24;18(3):e0283507. doi: 10.1371/journal.pone.0283507 (PMC10038266; doi:10.1371/journal.pone.0283507)
Supplement: S1 Table — (DOCX) [file pone.0283507.s001.docx]

**S1 Table. Cox regression models estimating the relationship between EFS score and OS in patients stratified by curative and palliative intent**

| Covariates | Bivariate models | | Multiple model | |
| --- | --- | --- | --- | --- |
|  | HR (95% CI) | p-value | HR (95% CI) | p-value |
| ***Curative intent (N=162)*** | | | | |
| EFS score  Age  Gender (female)  Cancer type  Breast  Prostate  Lung  Other  ECOG_dichotomised (2-4) | 1.20 (1.01; 1.43)  1.08 (1.01; 1.16)  0.46 (0.15; 1.40)  1  1.61 (0.10; 25.69)  54.11 (6.64; 440.78)  26.83 (3.00; 240.30)  0.05 (0.00; 5375.93) | **0.034**  **0.031**  0.172  0.737  **<0.001**  **0.003**  0.606 | 1.10 (0.89; 1.37)  1.06 (0.96; 1.18)  0.88 (0.25; 3.04)  1  1.52 (0.07; 31.83)  50.60 (5.43; 471.79)  12.54 (0.85; 184.12)  0.00 (0.00; NA) | 0.370  0.266  0.835  0.787  0.001  0.065  0.987 |
| ***Palliative intent (N=138)*** | | | | |
| EFS score  Age  Gender (female)  Cancer type  Breast  Prostate  Lung  Other  ECOG_dichotomised (2-4) | 1.15 (1.07; 1.23)  1.00 (0.97; 1.03)  1.02 (0.69; 1.50)  1  0.76 (0.35; 1.66)  1.20 (0.60; 2.40)  0.91 (0.46; 1.83)  1.87 (1.23; 2.83) | **<0.001**  0.795  0.932  0.493  0.606  0.799  **0.003** | 1.17 (1.06; 1.28)  1.00 (0.96; 1.03)  0.84 (0.53; 1.33)  1  0.61 (0.23; 1.61)  1.26 (0.58; 2.77)  0.95 (0.44; 2.05)  1.04 (0.60; 1.80) | **0.001**  0.821  0.453  0.322  0.560  0.892  0.895 |
