# Supplementary material for: Edmonton Frail Scale predicts mortality in older patients with cancer undergoing radiotherapy—A prospective observational study
Source: PLoS One. 2023 Mar 24;18(3):e0283507. doi: 10.1371/journal.pone.0283507 (PMC10038266; doi:10.1371/journal.pone.0283507)
Supplement: S2 Table — (DOCX) [file pone.0283507.s002.docx]

**S2 Table.** Sensitivity, specificity, and area under the curve (AUC) for different EFS cut-offs in relation to different numbers of mGA impairments

| mGA number of impairments | EFS  ≥1 | | EFS  ≥2 | | EFS  ≥3 | | EFS  ≥4 | | EFS  ≥5 | | EFS  ≥6 | | EFS  ≥7 | | EFS  ≥8 | | EFS  ≥9 | | EFS  ≥10 | | EFS  ≥11 | |
| --- | --- | --- | --- | --- | --- | --- | --- | --- | --- | --- | --- | --- | --- | --- | --- | --- | --- | --- | --- | --- | --- | --- |
|  | SE | SP | SE | SP | SE | SP | SE | SP | SE | SP | SE | SP | SE | SP | SE | SP | SE | SP | SE | SP | SE | SP |
| 0 | 1 | 0 | 1 | 0 | 1 | 0 | 1 | 0 | 1 | 0 | 1 | 0 | 1 | 0 | 1 | 0 | 1 | 0 | 1 | 0 | 1 | 0 |
| ≥1 | 0.86 | 0.71 | 0.90 | 0.57 | 0.96 | 0.44 | 0.98 | 0.35 | 0.99 | 0.28 | 1 | 0.25 | 1 | 0.23 | 1 | 0.21 | 1 | 0.19 | 1 | 0.18 | 1 | 0.17 |
| ≥2 | 0.63 | 0.93 | 0.71 | 0.96 | 0.81 | 0.85 | 0.89 | 0.74 | 0.95 | 0.65 | 0.97 | 0.57 | 1 | 0.54 | 1 | 0.49 | 1 | 0.44 | 1 | 0.43 | 1 | 0.41 |
| ≥3 | 0.46 | 1 | 0.51 | 1 | 0.62 | 0.96 | 0.72 | 0.90 | 0.80 | 0.83 | 0.87 | 0.78 | 0.92 | 0.74 | 0.93 | 0.69 | 0.94 | 0.63 | 0.96 | 0.60 | 1 | 0.59 |
| ≥4 | 0.29 | 1 | 0.32 | 1 | 0.39 | 0.99 | 0.49 | 0.99 | 0.60 | 0.97 | 0.71 | 0.94 | 0.80 | 0.91 | 0.82 | 0.86 | 0.85 | 0.80 | 0.91 | 0.78 | 1 | 0.76 |
| ≥5 | 0.20 | 1 | 0.22 | 1 | 0.27 | 1 | 0.34 | 1 | 0.44 | 1 | 0.54 | 0.99 | 0.62 | 0.97 | 0.68 | 0.94 | 0.82 | 0.90 | 0.86 | 0.87 | 1 | 0.85 |
| ≥6 | 0.16 | 1 | 0.18 | 1 | 0.22 | 1 | 0.27 | 1 | 0.35 | 1 | 0.43 | 0.99 | 0.53 | 0.99 | 0.58 | 0.96 | 0.71 | 0.92 | 0.82 | 0.91 | 1 | 0.89 |
| ≥7 | 0.10 | 1 | 0.12 | 1 | 0.14 | 1 | 0.18 | 1 | 0.23 | 1 | 0.29 | 1 | 0.36 | 1 | 0.38 | 0.97 | 0.56 | 0.96 | 0.68 | 0.95 | 0.86 | 0.94 |
| ≥8 | 0.04 | 1 | 0.05 | 1 | 0.06 | 1 | 0.07 | 1 | 0.10 | 1 | 0.12 | 1 | 0.15 | 1 | 0.17 | 0.99 | 0.27 | 0.99 | 0.32 | 0.98 | 0.36 | 0.98 |
| ≥9 | 0.01 | 1 | 0.01 | 1 | 0.01 | 1 | 0.01 | 1 | 0.02 | 1 | 0.02 | 1 | 0.03 | 1 | 0.03 | 1 | 0.06 | 1 | 0.09 | 1 | 0.14 | 1 |
| AUC  (95% CI) | 0.86  (0.79; 0.94) | | 0.88  (0.84; 0.92) | | 0.89  (0.85; 0.93) | | 0.90  (0.86; 0.93) | | 0.91  (0.88; 0.94) | | 0.92  (0.89; 0.95) | | 0.94  (0.91; 0.96) | | 0.92  (0.88; 0.95) | | 0.92  (0.87; 0.96) | | 0.93  (0.88; 0.98) | | 0.96  (0.94; 0.99) | |

Abbreviations: mGA, modified Geriatric Assessment; EFS, Edmonton Frail Scale; SE, Sensitivity; SP, Specificity
